# Current evidence to support the therapeutic potential of flavonoids in oxidative stress-related dermatoses

**DOI:** 10.1080/13510002.2021.1962094

**Published:** 2021-08-06

**Authors:** Dehai Xian, Menglu Guo, Jixiang Xu, Yang Yang, Yangmeng Zhao, Jianqiao Zhong

**Affiliations:** a Department of Anatomy, Southwest Medical University, Luzhou, People’s Republic of China; b Department of Dermatology, Affiliated Hospital of Southwest Medical University, Luzhou, People’s Republic of China

**Keywords:** Oxidative stress (OS), OS-related skin diseases, flavonoids, molecular mechanisms

## Abstract

**Background:**

Skin, as a crucial external defense organ, is more vulnerable to oxidative stress (OS) insult, reactive oxygen species (ROS)-mediated OS in particular. OS results from a redox imbalance caused by various extrinsic stimuli and occurs once the oxidants production overwhelming the antioxidants capacity, through mediating in DNA damage, lipid peroxidation (LPO), protein oxidation and a serial of signaling pathways activation/inactivation, thereby offering favorable conditions for the occurrence and development of numerous diseases especially some dermatoses, e.g. psoriasis, vitiligo, skin photodamage, skin cancer, systemic sclerosis (SSc), chloasma, atopic dermatitis (AD), pemphigus, etc. Targeting OS molecular mechanism, a variety of anti-OS agents emerge, in which flavonoids, natural plant extracts, stand out.

**Objectives:**

To discuss the possible mechanisms of OS mediating in dermatoses and summarize the properties of flavonoids as well as their applications in OS-related skin disorders.

**Methods:**

Published papers on flavonoids and OS-related skin diseases were collected and reviewed via database searching on PubMed, MEDLINE and Embase, etc.

**Results:**

It has been confirmed that flavonoids, belonging to polyphenols, are a class of plant secondary metabolites widely distributed in various plants and possess diverse bioactivities especially their potent antioxidant capacity. Moreover, flavonoids benefit to suppress OS via eliminating free radicals and mediating the corresponding signals, further excellently working in the prevention and management of OS-related skin diseases.

**Conclusion:**

Flavonoids have the potential therapeutic effects on oxidative stress-related dermatoses. However, more studies on specific mechanism as well as the dosage of flavonoids are needed in future.

## List of Abbreviations

AD: atopic dermatitis
Akt: protein kinase B
AP-1: activator protein-1
ARE: antioxidant reaction element
BCC: basal cell carcinoma
BHA: butyl hydroxyanisole
BHT: butyl hydroxytoluene
CAT: catalase
CD8+: cluster of differentiation eight positive
COX-2: cyclooxygenase-2
CPDs: cyclobutane pyrimidine dimers
DNCB: dinitrochlorobenzene
DPPH: 1-diphenyl-2-picrylhydrazyl
Dsg: desmoglein
ECM: extracellular matrix
EGCG: epigallocatechin gallate
ERKs: extracellular signal-regulated kinases
GSH: glutathione
GSH-Px: glutathione peroxidase
GSPs: grape seed proanthocyanidins
H_2_O_2_: hydrogen peroxide
HaCaT: human skin keratinocytes
HHD: Hailey-Hailey disease
HO-1: heme oxygenase
IκB: NF-κB inhibitory protein
IKK: nuclear factor kappa-B inhibitory protein kinase
IL-6: interleukin-6
iNOS: inducible nitric oxide synthase
IR: ionizing radiation
JAK-STAT: Janus kinase-signal transducer and activator of transcription
JNKs: c-Jun amino-terminal kinases
Keap1: kelch-like ECH-associated protein 1
LPO: lipid peroxidation
MAPKs: mitogen-activated protein kinases
MASI: melasma area severity index
MDA: malondialdehyde
MM: malignant melanoma
MMP: matrix metalloproteinase
mTOR: mammalian target of rapamycin
NADPH: nicotinamide adenine dinucleotide phosphate
NF-κB: nuclear factor kappa-B
NO: nitric oxide
Nrf2: nuclear factor-erythroid 2-related factor
OS: oxidative stress
PCs: proanthocyanidins
PG: propyl gallate
PI3 K: phosphoinositide-3-kinase
PKC: protein kinase C
PV: pemphigus vulgaris
RNS: reactive nitrogen species
ROS: reactive oxygen species
SCC: squamous cell carcinoma
SOD: superoxide dismutase
SSc: systemic sclerosis
TNF: tumor necrosis factor
UV: ultraviolet rays

## 1. Introduction

As the most external defense barrier of body, skin protects body against various harmful stimuli, so that it tends more possible to suffer from some exogenous attacks. Under normal conditions, the oxidant/antioxidant system in skin keeps a dynamic balance (also called *redox state*) that is essential to cellular physiological processes including intracellular signal transduction, cell growth, proliferation, aging and apoptosis [1]; however, this redox equilibrium may be broken by excessive reactive oxygen species (ROS) [e.g. superoxide anion (^•^O_2_^−^), hydroxyl free radical (^•^HO), singlet oxygen (^1^ O_2_), hydrogen peroxide (H_2_O_2_), etc.] or/and reactive nitrogen species (RNS) [e.g. nitric oxide (•NO), nitrogen dioxide (•NO_2_) and nitrite peroxide (^•^ONOO^−^), etc.], which further create oxidative stress (OS) and consequently encourage skin damage and dermatoses occurrence [2, 3]. OS, a state of ROS/RNS (ROS dominant) overproduction and/or antioxidant defense reduction, potentially facilitates molecular damage and a disruption of redox signaling [4]. As the major contributor, ROS are vital to the initiation and progress of OS. Among them, endogenous ROS is generated by mitochondrial respiratory chain, reaction catalyzed by nicotinamide adenine dinucleotide phosphate (NADPH) oxidase, nitric oxide synthase, and xanthine oxidase, as well as the inflammatory cells such as macrophages and eosinophils; the exogenous ROS mainly comes from the environment, such as ultraviolet rays (UV), ionizing radiation (IR), and so on. Once ROS generation far exceeds their elimination at the stimulation of environmental, biological and chemical factors, OS would occur and cause cutaneous cell/tissue injury, thereby leading to the development of numerous skin disorders such as psoriasis, vitiligo, skin photodamage, skin cancer, systemic sclerosis (SSc), chloasma, atopic dermatitis (AD), pemphigus and so on [5–9].

At present, many pharmaceutic products or agents targeting OS are employed in OS-related disorders recovery, like antioxidants [e.g. butyl hydroxyanisole (BHA), butyl hydroxytoluene (BHT), propyl gallate (PG), VitC or VitE, etc.], inhibitors of NADPH oxidases and others, among which natural plant extracts emerge as the optimal candidate owing to their powerful functions and few side effects. Flavonoids, as the typical representative, are particularly outstanding. They belong to polyphenols and are the common secondary metabolites widespread in various plants [10]. Until now, about 8,000 species of flavonoids have been identified and appear in the form of aglycones or in combination with glycosides and acetyls. They exhibit rare toxic and side effects but diverse properties including antioxidation, anti-inflammation and immunoregulation, antioxidative activity in particular, thereby being involved in various disorders prevention/treatment and spreading great application prospects [11–13]. Just because of their strong antioxidative effect, flavonoids these days have been applied in a variety of OS-related diseases especially in dermatoses by scavenging peroxyl radicals, inhibiting oxidase and combating OS via repairing damaged DNA/lipids/proteins, mediating related signal pathways and regulating cytokines release [14]. Thus, targeting OS, flavonoids would be effective in treating OS-related skin diseases and be hopeful for curing these dermatoses suffers.

## 2. Possible mechanisms of OS in cutaneous disorders

Currently, increasing evidence shows that OS is closely involved in many skin diseases. Physiological-level OS is regarded as self-defense mechanisms of body, whereas overwhelming OS may be greatly injurious. Substantial ROS trigger OS that in turn directly impairs cellular components or macromolecules (e.g. DNA, lipids, proteins, etc.) as well as mediating multiple signaling pathways, eventually leading to a series of skin disorders [15].

### 2.1. Oxidative damage to macromolecules in skin events

#### 2.1.1. DNA oxidative injury

ROS-caused oxidative damage may greatly induce DNA injury. It has been confirmed that overexposure to UV not only directly injures DNA, but also promotes ROS production in large quantities to provoke nuclear DNA oxidative damage even mutations [16]. Typically, accumulative UV-produced ROS facilitate skin photo-oxidative stress and p53 mutation, which results in apoptosis resistance and mitochondrial dysfunction, followed by irreparable DNA mutation and malignant proliferation in cutaneous cells, consequently contributing to a series of skin cancers, like squamous cell carcinoma (SCC), basal cell carcinoma (BCC) and malignant melanoma (MM) [17]. As one of p53 targets, pro-opiomelanocortin gene, coding precursor peptides of α-melanocorticoid-stimulating hormone and adrenocorticotrophic hormone, highly expresses after UV radiation and accelerates an increase in melanin, thus leading to the occurrence and progression of pigmentary dermatoses, chloasma in particular [18].

#### 2.1.2. Lipid peroxidation

High-level ROS induced by UV have the capacity to react with polyunsaturated fatty acids, therefore giving rise to lipid peroxidation (LPO) that is measured by peroxidation products (e.g. acrolein, phospholipid aldehydes and malondialdehyde (MDA), etc), further to impair phospholipids and mediate in immune/inflammatory response and gene expression [19, 20]. Results from imiquimod-induced psoriasis-like mice models showed that the level of LPO products (MDA) remarkably ascended, accompanied by antioxidant enzymes decrease after imiquimod application; in turn, the insufficient antioxidant enzymes triggered the bimolecular peroxidation of erythrocyte membrane further to aggravate the disease [21]. Oxidative damage to lipid alters the membrane permeability and stability, consequently contributing to direct lysis or death of cutaneous melanocytes that further spurs vitiligo initiation [22]. Besides, Abida et al. confirmed that ROS-induced LPO reaction and inflammatory cytokines would destroy the intraepidermal junction to arouse pemphigus vulgaris (PV) [23]. Similarly, it was discovered that not only an enhancement of LPO and a decline of antioxidant activity emerged from the plasma and red blood cell of PV patients [24], but also increased LPO and ROS would cause oxidative alterations in the structure of PV antibodies [desmoglein (Dsg) 1 and Dsg3], thus making it a potential for autoantibodies to induce spinous membrane dissolution [25]. In general, under the influence of various factors, LPO eventually facilitates the occurence and progression of skin diseases like vitiligo, photoaging, psoriasis and PV.

#### 2.1.3. Protein oxidation

Under OS state, highly ROS induce proteins dehydrogenation and produce protein free radicals, which are in turn converted into peroxide free radicals [26]. The free radicals directly attack the protein main chain and amino acid residual side chain to produce protein carbonyl derivatives that encourage the loss of protein function and the inhibition of enzyme activity, consequently contributing to cutaneous cells injury/aging and various skin diseases [27]. OS-damaged DNA repair proteins would prevent DNA repair and induce DNA mutations to trigger skin cancer initiation, SCC, BCC and MM in particular [28, 29]. Likewise, Spencer et al. discovered an accumulation of hydrogen peroxide (H_2_O_2_) in vitiligo epidermis that accelerated the oxidation of vitiligo epidermal ACTH and β-endorphin, thus proving that H_2_O_2_ influenced pigmentation via epidermal proopiomelanocortin peptides redox homeostasis [30]. Under the state of OS, H_2_O_2_ could promote melanosome protein breakdown and calcium oxide protein production that creats an imbalance in calcium homeostasis, therefore affecting melanin synthesis and initiating vitiligo [31]. Additionally, in pemphigus, excessive ROS impel the oxidative modification of proteins such as Dsg1 and Dsg3 that stimulate the immune system to produce autoantibodies and cause acantholysis [25].

### 2.2. Key signaling pathways involved in OS in skin events

Apart from OS damage to macromolecules, growing evidence supports that multiple critical signaling pathways are involved in ROS-induced OS in dermatoses, mainly comprising nuclear factor-erythroid 2-related factor (Nrf2), mitogen-activated protein kinases (MAPKs), Janus kinase-signal transducer and activator of transcription (JAK-STAT), nuclear factor kappa-B (NF-κB) and phosphoinositide-3-kinase (PI3 K)/protein kinase B (Akt). Through mediating in above signal pathways, OS pervades a variety of pathological molecular events i.e. inflammation, injury and tumourigenesis in skin, eventually triggering AD, skin photodamage/ photoaging and skin photocarcinogenesis.

#### 2.2.1. OS and Nrf2 signaling pathway

Nrf2 is a master regulator in OS to protect skin cell/tissue from oxidative damage. Normally, Nrf2 partakes in skin homeostasis maintenance by bound to kelch-like ECH-associated protein 1 (Keap1). In OS condition, excessive ROS could prevent Nrf2 nuclear translocation and spur it inactivation. The inactivated Nrf2, whereas, fails to enhance the capacity of antioxidant enzymes/antioxidants and facilitates more ROS accumulation, further exciting MAKPs and NF-κB signaling pathways and encouraging dermal matrix degradation, tumour suppressor gene inactivation and cell apoptosis [32, 33]. An *in vitro* study on SSc revealed a notable down-regulation of Nrf2 appearing in SSc fibroblasts, which facilitated glutathione (GSH) decline and substantial ROS generation, followed by the activation of MAPKs pathway, the proliferation of fibroblasts as well as the formation of collagen [34]. In vitiligo, inactive Nrf2 signal was inefficient to protect human melanocytes from H_2_O_2_-induced OS damage and thus seldom stopped melanocytes apoptosis via mediating its downstream antioxidant gene heme oxygenase-1 (HO-1) [35]. Therefore, Nrf2 activation has become an important target in protection against OS-related dermatoses like skin photodamage, SSc and vitiligo.

#### 2.2.2. OS and MAPKs signaling pathway

The MAPK family kinases, primarily involving extracellular signal-regulated kinases (ERKs), c-Jun amino-terminal kinases (JNKs) and p38 kinase (p38), modulate the transcriptional cascades to mediate stress responses in cells and impel extracellular signals transduction into intracellular environment [36].

In most cases, ROS facilitate the phosphorylation and translocation of ERKs, JNKs and p38 kinase; whereas, the activation of these pathways further exacerbates OS as well oxidative damage that would initiate and aggravate skin diseases. For instance, it was found an apparent increase of ERK, p38 MAPK and JNK activation in the impaired skin of psoriasis [37]. Substantial UV-induced ROS from skin efficiently provoke the ERK, JNK, and p38MAPK pathways to spur diverse cytokines secretion by sensitizing the activator protein (AP)-1 and upregulating the expression of the cyclooxygenase-2 (COX-2) gene, further contributing to a series of molecular events particularly immune suppression, inflammatory response and angiogenesis in skin; these events offer the guarantee for photoaging acceleration and tumour cell infiltration, thereby leading to the invasion and metastasis of light-related skin cancers especially SCC [38]. Simultaneously, AP-1 drives the enhancement of cathepsin K and matrix metalloproteinase (MMP), and the degradation of dermal collagen, elastin and matrix, hence aggravating skin photoaging [39]. Besides, Boilan et al. considered that OS could increase p38 MAPK phosphorylation to spur the internalization of Dsg3 and the dissolution of epidermal spinous cells [40]; furthermore, the excited p38MAPK pathway deeply affect the formation of blister in PV, thus targeting this pathway would be quite promising for pemphigus recovery.

#### 2.2.3. OS and NF-κB signaling pathway

NF-κB, a nuclear transcription factor, positively works in a series of skin physiological and pathological activities, such as inflammation, proliferation, senescence and apoptosis. As the crucial signal of inflammation and apoptosis, NF-κB frequently highly expresses in some OS-related inflammatory dermatoses, psoriasis as a typical case. A study of psoriasis showed that NF-κB in high-profile emerged from psoriatic keratinocytes [41]. Additionally, OS could activate the NF-κB pathway and facilitate the release of histamine by inflammatory cytokines, further to exacerbate the progression of AD [42]. As the critical downstream target of MAPKs pathway, NF-κB/p65 signaling crossed each other and worked together to participate in skin photoaging and photocarcinogenesis [43].

#### 2.2.4. OS and JAK/STAT pathway

The JAK/STAT pathway frequently mediated by ROS is one of the ways to transmit extracellular signals and get involved in regulation of OS [44]. Under the stimulation of ROS from skin, JAKs is firstly activated, followed by the activation of STAT protein, which further excites or suppresses the target genes and regulates the transcription of various cytokines in skin [45]. Several studies of psoriasis have shown that ROS-induced STAT3 activation would encourage cell hyper-proliferation and elevate the production of interleukin-6 (IL-6), IL-8, IL-23 and IL-17, which in turn provoke Th17 cell and STAT3 signaling pathway, gradually switching towards persistent inflammation [46]. Chen et al. meanwhile discovered that OS-induced IL-15 trans-presentation in epidermal keratinocytes benefited the activation of cluster of differentiation eight positive (CD8+) T cells in vitiligo via activating JAK/STAT pathway [47]. Apart from, the study on xeroderma pigmentosum cells discovered that UV irradiation could stimulate human fibroblasts to generate ROS that in turn triggered DNA damage and STAT1 phosphorylation, which further elevated the expression of MMP-1, potentially initiating skin photoaging and skin tumours [48].

#### 2.2.5. OS and PI3K/Akt signaling pathway

PI3K/Akt extensively exists in cells, especially in cutaneous cells, to regulate normal cellular activities [49]. Normally, ROS directly activate PI3K and then excite Akt via interaction with related molecules, which is beneficial to the transcription of target genes and the physiological activity of cells [50]. However, hyperactive PI3K/Akt could stimulate Nox activation that in turn facilitates an overproduction of ROS; whereas continuous ROS exposure would confer a potential of mutation and be considered as a major contributor to some disorders like skin cancer and aging [51]. Moreover, excessive ROS from UV activate PI3K/Akt/mTOR (mammalian target of rapamycin) pathway to initiate several cutaneous diseases like psoriasis, MM and chloasma, therefore considering this pathway as a pivotal target for these dermatoses treatment [52]. In psoriasis, the PI3 K/Akt signaling pathway is discovered to be over-activated that encourages the phosphorylation of mTOR and promotes the proliferation of keratinocytes in psoriasis lesions [53]. The melanoma models also showed that activated Akt is implicated in MM and its invasiveness, accompanied with the elevation of mTOR, peroxides and angiogenesis [54]. In addition, an available experiment on chloasma suggested that UV-induced ROS greatly elevated the activity of tyrosinase by stimulating PI3K/Akt signaling pathway, thereby facilitating the increase of melanin [55]. Hence, blockage of this pathway to some extent would be a favourable approach for above dermatoses therapy.

**Figure 1.**
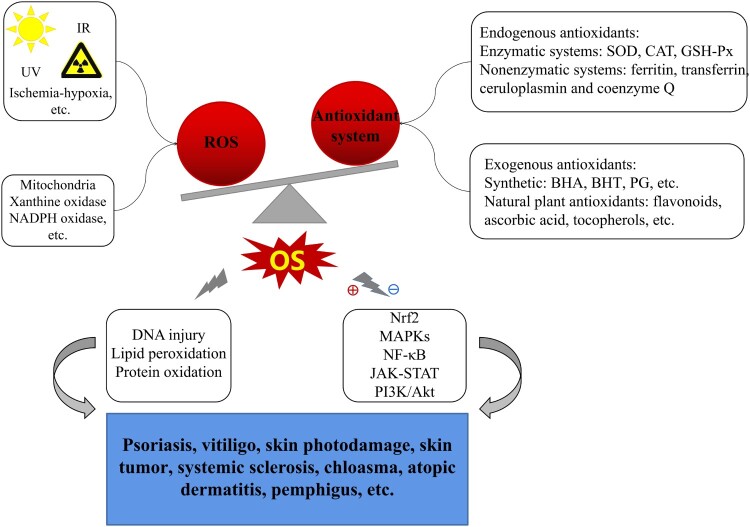
Possible mechanisms of OS mediating in skin diseases. Exogenous insults (UV, IR, ischemia-hypoxia, etc.) and/or endogenous factors (oxidase, metabolism) induce ROS overproduction, far beyond of antioxidant defense capability triggering OS occurrence; OS, then, facilitates macromolecules damage (including DNA injury, LPO, protein oxidation) and mediates in several related signaling pathways (e.g. Nrf2, MAPKs, NF-κB, JAK-STAT, PI3K/Akt), eventually resulting in various dermatoses such as psoriasis, chloasma, vitiligo, skin photodamage, skin tumour, SSc, AD, pemphigus and so on. Notes: ⊕indicates ‘activation’; ⊖indicates ‘inhibition or suppression’.

The above-mentioned possible mechanisms of OS in cutaneous disorders are summarized in Figure 1.

## 3. Plant-based antioxidants flavonoids and OS-related dermatoses

Redox homeostasis of cutaneous microenvironment is principally guarded by antioxidant defense system that comprises endogenous antioxidants and exogenous antioxidants. Endogenous antioxidants in enzymology are divided into enzymatic defense and nonenzymatic counterpart. The former contains superoxide dismutase (SOD), catalase (CAT) and glutathione peroxidase (GSH-Px); while the latter covers ferritin, transferrin, ceruloplasmin, and coenzyme Q, etc. [56]. Exogenous antioxidants primarily involve synthetic antioxidants (BHA, BHT, PG, etc) and natural plant counterparts. Natural plant antioxidants, e.g. phenolics flavonoids, ascorbic acid and tocopherols, derived from vegetables, grains and fruits, are increasingly in the spotlight for their powerful antioxidant function and few side adverse [57]. As a perfect example, flavonoids especially deserve more attention in skin field.

### 3.1. Characteristics of flavonoids

#### 3.1.1. Structure, distribution and classifications of flavonoids

Flavonoids constitute a large group of plant secondary metabolites ubiquitously occurring in fruits, vegetables, nuts, flowers and grain seeds, etc. [10]. As a diverse range of bio-active plant/food compounds, flavonoids in chemical structures are composed of 15 carbon skeletons, with a C6-C3-C6 framework formed by two aromatic rings (A and B) through a 3-carbon chain of oxygen-containing heterocyclic C ring [58, 59]. Based on the functional groups on the ring, the generic structure and the degree of unsaturation and oxidation of the C ring, flavonoids can be chiefly classified into six subgroups, namely flavones, flavonols, flavanones, flavanols, isoflavones and anthocyanins [60]. These flavonoids have been researched to treat a variety of skin diseases, including psoriasis, vitiligo, skin photodamage, skin cancer, SSc, chloasma, AD, and pemphigus; among them, *epigallocatechin gallate (EGCG), luteolin, apigenin, quercetin, kaempferol, fisetin, silymarin, apigenin, and proanthocyanidins* (*PCs*) have been studied extensively.

The following table (Table 1) shows the typical classification, structure and representatives of flavonoids.

**Table 1.** Classification and structure of flavonoids.

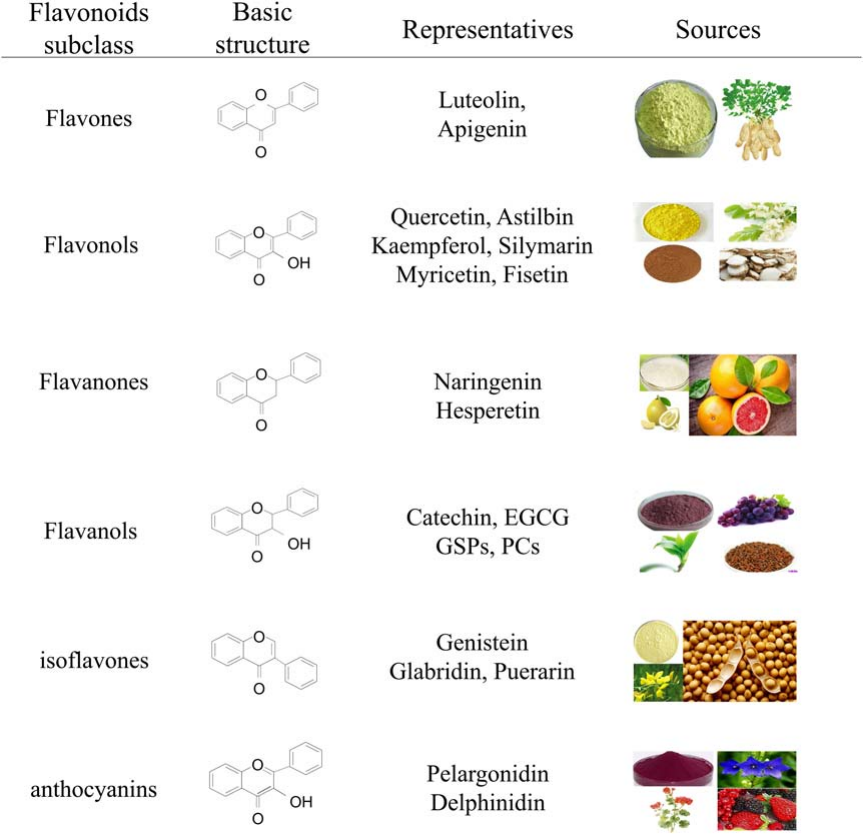

#### 3.1.2. Metabolism and bioavailability of flavonoids

The metabolism of flavonoids in the body mainly consists of hydrolysis, binding, lysis and oxidation, primarily occuring in the intestine and liver [61]. Flavonoids are commonly present in food in the form of glycosides [62]. Some of the aglycones are directly absorbed by the intestinal tract, while others are lysed to produce various phenolic acids, which are further transported to the liver to form polar compounds and water-soluble metabolites [63]. These metabolites are largely excreted through urine, but part of them with bile return to intestine where they are hydrolyzed to glycosides and absorbed into the blood, forming a liver-intestinal circulation [64].

Although flavonoids have lots of health benefits, their bioavailability is generally low, with a significant difference depending on their specific subclasses [65]. Many factors affect the bioavailability of flavonoids ingested in the diet, involving the absorptivity, chemical molecular structures, and roles of glycosylation and esterification [66]. Currently, some advanced techniques, e.g. nano-dosing system, micro-capsulation, and enzymatic-methylation modification, are developed and employed to change their bioavailability [64].

#### 3.1.3. Biological activities of flavonoids

Numerous studies have demonstrated that flavonoids exhibit diversified bioactivities compraising antioxidation, anti-inflammation, anti-mutation, anti-carcinogenesis, anti-aging and immunomodulation [10]. With multiple functions and few side adverses (even application in pregnants), they serve humankind as rescuers, not only greatly benefiting people health and dietary ingredients, but also lowering the risk of health problems, like age-related degenerative diseases, cardiovascular disease, OS-related dermatoses and cancers. However, recently more studies focus on the antioxidant activity of flavonoids owing to their powerful capacity of free radical scavenging [67]. To date, flavonoids have been developed against OS to treat OS-related dermatoses by protecting macromolecular substances and regulating redox signaling pathways.

### 3.2. Favourable role of flavonoids in OS-related dermatoses

OS status in the skin microenvironment affords profitable conditions for the initiation and progression of dermatoses. It has been demonstrated that OS is closely implicated in the occurrence and development of psoriasis, vitiligo, skin photodamage, skin cancer, SSc, chloasma and AD, etc. [15]. Through damage to macromolecular substances (e.g. DNA, lipids, and proteins) and mediation in several pathways (including Nrf2, MAPK, JAK-STAT, PI3 K/Akt and NF-κB), OS provokes and aggravates cutaneous disorders attacks. Thanks to their potent antioxidant activity, flavonoids are quite available for controlling dermatoses by directly scavenging ROS to repair damaged macromolecules and by indirectly reducing ROS via upregulating antioxidant enzyme activities (SOD, CAT, GSH-Px) and modulating OS-mediated pathways. The following parts detailedly describe the role of flavonoids in several representative skin diseases.

#### 3.2.1. Flavonoids in psoriasis

Psoriasis is a frequent chronic inflammatory disease associated with immune, environmental and genetic factors, which has been confirmed that OS is implicated in the pathogenesis of psoriasis [2]. As the sponsor of OS in psoriasis, ROS from skin induce the expression of inducible nitric oxide synthase (iNOS), MDA and nitric oxide (NO), while inhibit the activity of SOD, CAT and GSH-Px, further activating Th1/Th17 cells and keratinocytes through stimulation of MAPK, NF-κB and JAK-STAT pathways [68, 69], thereby producing the cascade of inflammatory factors. These inflammatory mediators in turn irritate T cells and mast cells, facilitating keratinocytes proliferation, neutrophils recruitment, angiogenesis and persistent inflammation in skin [41].

Recently, numerous studies have revealed that several subclasses of flavonoids as bioactive compounds are extremely useful in controlling psoriasis. *Glabridin*, an isoflavone (one of flavonoids) from licorice roots, possesses multiple interesting properties covering anti-oxidant and anti-inflammatory activities [70]. In psoriasis-like mice models, *glabridin* could enhance the activities of SOD, CAT and GSH, and lower the levels of MDA and pro-inflammatory cytokines to mitigate the inflammation and histopathological alterations i.e. a *glabridin-*dose-dependent reduction of epidermal thickness and lymphocyte infiltration in the dermis [71]; therefore, *glabridin* is an effective anti-psoriasis bioactive compound. Meanwhile, *luteolin*, plant derived flavonoids, in a dose-dependent manner suppressed the iNOS protein produced by RAW264.7 cells after LPS stimulation, which was beneficial to alleviation of imiquimod-induced psoriasis-like skin lesions in BALB/c mice [72]. A preclinical evidence supports that *EGCG* from flavanols can significantly attenuate clinical symptoms (like pruritus) and pathologic features (like epidermal hyperplasia and dermal inflammatory cells infiltration) on imiquimod-induced psoriasis-like mice via restraining OS and inflammation-related pathways especially Akt signaling pathway [73]. Owing to its potent antioxidant and anti-inflammatory effects on particulate matte-exposed human skin keratinocytes (HaCaT), *afzelin* (kaempferol-3-O-rhamnoside) has been used to control air pollution-induced psoriasis through suppressing p38 MAPK and AP-1 phosphorylation to reduce intracellular ROS production [74]. In addition, several experiments on psoriasis-like models have confirmed that flavonols (e.g. *quercetin* and *astilbin*) would be greatly potential to psoriasis healing via inhibiting Th17 cell differentiation, enhancing Nrf2 signal and blocking NF-κB or JAK/STAT3 pathways further to eliminate ROS and inflammation [75, 76]. Other flavonoids particularly *delphinidin* exhibits powerful anti-psoriatic effects *in vivo* and *in vitro* on a psoriasis-like model of Balb/c mice as well as a reconstructed psoriatic skin equivalent via PI3K/mTOR pathway; most notably, topical *delphinidin* distinctly decreased hyperproliferation and epidermal thickness [77, 78]. In an *in vivo* study, *isoflavone* extracts were topically applied on the mice prior to IMQ application, consequently which exhibited a significant decrease in trans-epidermal water loss, erythema and ear thickness, in that *isoflavone* effectively enhancing surface skin hydration and reducing inflammatory cells infiltration [79]; the same experiment *in vitro* showed *isoflavone* extracts remarkably prohibited the activation of MAPK, NF-κB and JAK/STAT in normal epidermal keratinocytes after induction with IL-22, IL-17A and tumour necrosis factor (TNF)-α; these together indicated that *isoflavone* extracts would be greatly potential to psoriasis treatment [79].

In brief, owing to their abilities of scavenging free radicals, LPO and modulating multiple redox signaling pathways, such as NF-κB, JAK/STAT3, Nrf2, p38 MAPK, flavonoids are proposed as a prospective antioxidant and anti-psoriatic agent for psoriasis recovery through arresting OS damage.

#### 3.2.2. Flavonoids in vitiligo

Vitiligo is a complex disease characterized by reduction in melanin pigment of skin resulting from abnormal melanocyte function. Although the etiology of vitiligo keeps unclear, overwhelming evidences support that OS is a major contributor to the decrease of melanocytes, thus provoking the onset and development of vitiligo [80]. Both exogenous and endogenous stimuli exacerbate melanocyte stress, leading to excessive H_2_O_2_ generation [81]; whereas overproduction of H_2_O_2_ triggers OS to impair Nrf2 signal activation in vitiligo melanocytes. OS not only decreases Nrf2 nuclear translocation and transcriptional capacity of vitiligo melanocytes but also lowers HO-1 expression, which would eventually destroy the synthesis of melanin [82]. Meanwhile, ROS accumulation and membrane peroxidation often emerge from melanocytes and keratinocytes in the uninvaded parts of vitiligo [83]. Besides, cytokines containing IL-1, IL-6 and TNF greatly enhance IL-8 expression in melanocytes, further inducing OS and ultimately contributing to the apoptosis of keratinocytes and melanocytes in vitiligo [84].

Targeted the mechanism of OS in vitiligo, a serial of flavonoids subgroups are promising in management of vitiligo. *Quercetin* as a potential agent for vitiligo also presents a cytoprotection against H_2_O_2_ and dramatically attenuates H_2_O_2_-mediated effects in vitiligo melanocytes [85]. Similarly, Ning et al. found that *EGCG* could protect human epidermal melanocytes against OS damage through scavenging excessive H_2_O_2_-induced ROS in a concentration-dependent manner [86]. Moreover, flavonoids from *Ginkgo biloba* extracts prominently eliminated ROS accumulation or LPO and availably protected vitiligo melanocytes against OS-induced apoptosis through activation of Nrf2 pathway and its downstream antioxidant genes [87]. Additionally, *afzelin* could not only prevent H_2_O_2_-induced apoptosis, LPO and ROS production in melanocytes, but also enhance the expression of Nrf2, HO-1 and CAT, therefore demonstrating that *afzelin* would be effective in the prevention of OS-induced melanocytes damage and the control of vitiligo through mediating Nrf2 pathway [88]. *Baicalein*, a kind of flavonoids, could strengthen melanocytic antioxidant defense, ameliorate mitochondrial dysfunction and mitigate cellular impairments in vitiligo via activating Nrf2 signaling pathway and enhancing HO-1 expression [89]; it, simultaneously, reduced intracellular ROS production in vitiligo skin and inhibited p38 MAPK pathway activation to stop OS invasion and protect melanocytes from oxidative damage [90]; therefore, *baicalein* would be promising for vitiligo. Other flavonoids like *apigenin* has a protective effect on dopamine-induced apoptosis of melanocytes; it protects melanocytes from OS insult via eliminating the accumulation of ROS and inhibiting the activation of OS-related pathways like JNK, p38 MAPK and Akt [83]; thus, *apigenin* may be considered as a potential candidate for the treatment of vitiligo, especially contact/occupational vitiligo. Besides, *luteolin*, apparently blocks TNF and IL-1 to release IL-8 in melanocyte, indicating an antioxidant and anti-inflammatory effect of *luteolin* on early vitiligo [84]. Nowadays, it is consistently considered that the combined use of flavonoids with psoralen UVA or trimethylpsoralen would be a desired vehicle for vitiligo treatment [91].

Thus, flavonoids, with strong antioxidant and anti-inflammatory activity, is hopeful to be potentially applied in the management of vitiligo.

#### 3.2.3. Flavonoids in skin photodamage

Skin photodamage is a specific injury in skin tissue directly or indirectly caused by a long-term or cumulative UV exposure. At present, convincing evidence reveals that UV-induced ROS overproduction and Nrf2 inactivation are major players in pathogenesis of skin photodamage. Excessive ROS from UV inactivate Nrf2 and prohibit Nrf2/antioxidant reaction element (ARE) signaling pathway as well as provoke other pathways (e.g. MAPKs, NF-κB and PI3 K/Akt), followed by a descend in the activity of antioxidant enzymes like HO-1, the disruption of antioxidant defense system and the deterioration of oxidative damage; further spoiling cutaneous cells and tissues, and resulting in skin photodamage or photoaging [92].

Flavonoids are classified as polyphenols that exert powerful photoprotective and anti-aging effects on several *in vitro* and *in vivo* photodamage-like models through their antioxidant and anti-inflammatory bioactivities. *Naringenin* is considered as a novel promising way for skin photodamage treatment; it, on one side, reduces the consumption of endogenous antioxidants through regulating the production of cytokines and scavenging free radicals, on the other side functions as an antioxidant in UVB-induced skin damage via inhibiting the production of free radicals [93]. Owing to its high capacity of scavenging free radicals, *silymarin,* rich in flavonoids, displays a protection against UVB-induced OS damage and obviously relieves UVB-irradiated sunburn. Studies showed that supplement of *silymarin* in the drinking water to SKH-1 hairless mice greatly mitigated UV-caused skin edema or erythema as well as the production of H_2_O_2_ in the epidermis and dermis [94]. Meanwhile, external use of *EGCG* (1mg/cm^2^ skin area) on human skin before UVB exposure could significantly attenuate UVB-induced cutaneous erythema, inflammatory leukocytes infiltration and myeloperoxidase activity, the possible mechanism relating to *EGCG* inhibition of UVB-induced ROS production and leukocyte infiltration [95]. In addition, *quercetrin*–the common flavonoid could reduce ROS generation in skin keratinocyte cells and restore CAT activity, further to inhibit UVB-induced cutaneous damage and apoptosis *in vitro* and *in vivo*, thereby verifying that quercitrin plays a photoprotective role in UVB-induced oxidative damage to skin [96, 97]. *Apigenin*, in the same way, availably protects keratinocytes against UVB-induced damage by restoring autophagy, inhibiting apoptosis/cell death and up-regulating DNA-damage response proteins, suggesting *apigenin* as a promising application in skin photodamage [98]. It was discovered that *fisetin*, a kind of flavonol existing in fruits and vegetables, has a favourable photoprotection against UVB-induced damage to skin fibroblasts and dermal collagen through decreasing intracellular ROS and NO production via mediating in multiple signaling pathways including MAPKs, NF-κB and PI3K/Akt [99]. Simultaneously, *myricetin* could remarkably reduce H_2_O_2_ production, inhibit LPO and block JNK pathway, further to alleviate UVB-induced damage in keratinocytes [100]. Apart from that, dietary intake of *PCs* (0.2 and 0.5% w/w) greatly lightened the acute or chronic UVB-irradiated damage to mice, along with the enhancement of GSH-Px, CAT and GSH, as well as the diminishment of LPO, hydrogen peroxide, protein oxidation and nitric oxides in mice serum, potentially being attributed to the suppression of ERK1/2, p38 proteins and NF-κB /p65 phosphorylation and the downregulation of COX-2, iNOS and MMP-9 genes expression in the presence of *PCs* [101, 102].

Above studies all suggest that flavonoids do well in skin photodamage and thus may be added to skin care products, especially sunscreens as antioxidants. However, the long-term effects and underlying mechanisms of flavonoids in skin photodamage should be further investigated, in order to ensure the optimal photoprotective effectiveness.

#### 3.2.4. Flavonoids in skin cancer

Skin cancer mainly includes three types, namely MM, BCC and SCC. BCC and SCC are assigned to non-melanoma skin cancer that originates from keratinized epithelial cells, while MM is the deadliest cutaneous cancer derived from melanocytes that are found in the basal layer of the epidermis. Exposure to UV for a long time could induce persistent ROS-mediated OS status. OS disturbs the homeostasis of skin cells and facilitates accumulative DNA damage and DNA repair related enzymes depletion that encourages p53 mutation of cutaneous cells, further prompting proto-oncogenes activation and tumour suppressive genes inactivation in keratinocytes or melanocytes [103]. As a result, malignant cutaneous cells would escape from ROS-induced apoptosis and proliferate out of control, eventually skin cancer like MM and SCC arising [104].

Flavonoids, e.g. *EGCG*, *silymarin*, *luteolin*, and *PCs* have been demonstrated to have the ability to attenuate DNA impairment and prohibit OS via lowering ROS generation, arresting LPO and up-regulating antioxidant enzymes, thereby be considered as a prospective therapeutic vehicle in skin cancers [105, 106]. *Grape seed proanthocyanidins* (*GSPs*) affords to prevent the occurrence of UV radiation-induced skin cancers through scavenging free radicals, diminishing the depletion of antioxidant enzymes (e.g. GSH-Px, CAT, SOD), reducing lipid/protein oxidative damage and inactivating MAPK and NF-κB pathways [107, 108]. Basing on its potent anti-OS, anti-mutation and anti-carcinogenesis properties, *luteolin* directly inhibits protein kinase C (PKC) ϵ/Src activities and prevents keratinocytes from UVB-induced DNA damage to positively combat skin cancers through attenuating the expressions of COX-2, AP-1 and NF-κB as well as their upstream signals (e.g. MAPKs and Akt) and suppressing UVB-induced OS and cyclobutane pyrimidine dimers (CPDs) formation [109, 110]. *Silymarin* vigorously upsets the balance of carcinogen metabolism and gene expression through inhibition of OS insult, consequently retarding the progress of skin cancers. Previous studies revealed that topical application of *silymarin* in UVB-induced mice skin carcinogenesis models availably prevented UVB-induced DNA damage, apparently restrained tumour incidence/multiplicity/volume and upregulated p53, thus offering strong protection against UVB-induced carcinogenesis, possibly through its substantial antioxidant activities [111]; further evidence supports that *silymarin* highly inhibited terephthalic acid-induced skin edema, epidermal LPO, myeloperoxidase activity and antioxidant enzymes depletion (SOD, CAT, and GSH-Px) in the epidermis to stop tumour promotion, confirming the anti-tumour-promoting effects of *silymarin* in non-melanoma skin cancers [112, 113]; *silymarin* also effectively controled NO production and the expression of ERK1/2, NF-κB/p65 and p38 by suppressing UVB-induced iNOS activity and detering the degradation of NF-κB inhibitory protein (IκB) α and activation of nuclear factor kappa-B inhibitory protein kinase (IKK) α [114]; thus above studies provide considerably reliable proof for *silymarin* in the treatment of cutaneous carcinoma via arresting OS. By inhibition of NF-κB activity, besides, *EGCG* can reduce several inflammatory enzymes/cytokines release, (including MMP, iNOS, COX-2, IL-6 and IL-8) to arrest melanoma survival, growth and development [115]. Compared with untreated animals, topical treatment with *EGCG* hydrophilic ointment on the skin of SKH-1 hairless mice significantly delayed the development of UVB-induced skin tumours [116].

Anyhow, more and more reports have shown that polyphenols containing flavonoids powerfully protect skin from the risk of UV-induced skin cancers via mitigating cutaneous inflammation, OS, LPO and DNA damage [117]. Therefore, flavonoids would be a potential alternative for skin cancers, particularly UV-induced cutaneous carcinoma.

#### 3.2.5. Flavonoids in systemic sclerosis

SSc, an immune-mediated systemic disease, is featured by microvasculature damage and progressive fibrosis of skin as well multi-visceral organs. The pathological characteristics mainly manifest as extracellular matrix (ECM) synthesis increase and decomposition decrease along with substantial ECM deposition in the skin and other organs, notably a significant enhancement of I type collagen [118].

Recent studies show that ROS-induced OS is a major contributor to the onset and progression of SSc [119, 120]. Large amounts of ROS generation in SSc could spoil endothelial cells and fibroblasts via mediating extracellular and intracellular oxidative processes [121]. Increased ROS not only promote the gene expressions of collagen type I and the synthesis of plasminogen activator inhibitor and tissue inhibitor of metalloproteinase, but also reduce ECM degradation, thereby resulting in tissue fibrosis and dysfunction [119]. Moreover, microvascular dysfunction facilitates tissue hypoxia and reperfusion injury, so creating massive free radicals that in turn impair vascular endothelium, further exacerbating vasoconstriction disorders.

Currently, growing proofs have indicated that flavonoids as the potent antioxidant are expected to become a profitable approach to SSc recovery. *Kaempferol*, belonging to natural flavonoids, exhibits excellent antioxidant activity. Relevant experiments *in vivo* and *in vitro* demonstrated that *kaempferol* could apparently inhibit mRNA expression of Nox2, suppress the release of inflammatory/pro-fibrotic cytokines and reduce the infiltration of inflammatory cells in *bleomycin*-induced dermal fibrosis-like OKD48 (Keap1-dependent Oxidative stress Detector, No-48-luciferase) mice [122]; meanwhile, oxidant-induced ROS accumulation and apoptosis in SSc fibroblasts fell off in the presence of *kaempferol*, implying that *kaempferol* would be a favourable alternative for skin fibrosis in SSc via attenuating OS, inflammation and oxidative damage [122]. Other reports have shown that *ginkgo biloba* extract-derived flavonoids may prevent the adhesion of endothelial cells stimulated by inflammatory cytokines to human monocytes through reducing intracellular ROS formation; it, in addition, reduce the frequency of Raynaud´s attacks in SSc [123, 124]. Above all, flavonoids give a great hope to patients who suffer from SSc.

#### 3.2.6. Flavonoids in chloasma

Chloasma, a facial irregular brown or grayish-brown pigmentation, often prefers female living in intense UV radiation areas [125]. Recently, it is widely considered that OS is responsible for the etiology-pathogenesis of chloasma. Results from the *in vitro* experiment revealed that the balance of anti-oxidant/oxidant was disrupted and the OS markers significant elevated in melasma with an abnormal increase in MDA, NO, Cu/Zn-SOD and GSH-Px and a decrease in protein carbonyl [126]. Besides, UV-produced free radicals could directly excite PI3K/Akt signaling pathway to activate NF-κB signal, which would promote the transcription of iNOS in keratinocyte to produce NO, further enhancing tyrosinase activity in melanocytes and ultimately increasing melanin formation [55, 127]; more importantly, it was discovered that OS indicators (like MDA, NO, ROS) positively correlated with melasma area severity index (MASI) in chloasma [55], further confirming the catalytic role of OS in chloasma pathogenesis.

Owing to the OS-mediated pathogenesis of chloasma, natural antioxidants flavonoids tend to be favourable in chloasma treatment. *Myrica rubra* fruit extracts, rich in *kaempferol* and *quercetin* with little cytotoxicity, could effectively inhibit melanin synthesis, lower tyrosinase activity and down-regulate the expression of microphthalmia transcription factor and tyrosinase-related protein 1 through clearing 1, 1-diphenyl-2-picrylhydrazyl (DPPH) free radicals and stopping ROS production, therefore, indicating that these compounds would be safe and effective in treating pigmentary skin diseases, chloasma in particular [128]. Meanwhile, Handog et al. through a randomized, double-blind, placebo-controlled trial drew a conclusion that PCs, a kind of flavonoids, exhibited an obvious improvement in MASI and an apparent drop in pigmentation after a 8-week treatment on women with chloasma [129]. Similarly, a clinical trial in female chloasma patients proved that three-time daily administration of *pycnogenol* from a *Pinus pinaster* bark extract containing flavonoids evidently decreased the average melasma area and pigmentary intensity possibly attributed to its role of photoprotection from UV and resistance to UV-induced OS [130]. Apart from that, *silymarin*, extracted from the seed of the milk thistle plant, has strong antioxidant and photoprotective effects to suppress UV-induced OS and inflammation; it could remarkably reduce the production of melanin through inhibiting the L-dopa oxidation activity of tyrosinase, preventing OS damage, and blocking UV-induced NF-κB pathway activation [127, 131]. In a randomized, double-blind controlled trial of 96 patients with chloasma, Altaei et al. showed that *silymarin* at a different dose was topically administrated to patients′ affected areas twice daily for 4 weeks; it turned out that *silymarin* eliminated skin pigmentation of chloasma patients in a dose-dependent manner, possibly being associated with antioxidant activity of *silymarin* via mitigating OS [132].

At present, there are relatively few studies about flavonoids in the treatment of chloasma basing on their antioxidant effect, and the specific therapeutic mechanism still keeps unclear. Therefore, it is necessary to carry out further studies on them.

#### 3.2.7. Flavonoids in atopic dermatitis

AD is a chronic recurrent inflammatory skin disease caused by genetic susceptibility, immune dysfunction and skin barrier defects. In recent years, emerging evidence supports that OS is a critical contributor to the pathogenesis of AD. OS induced by environmental, physiological or psychological factors encourages skin barrier dysfunction or immune abnormality, ultimately leading to the occurrence or exacerbation of AD [133]. Via activating the NF-κB pathway, OS could not only promote gene expression and antioxidant enzyme synthesis, but also induce the release of inflammatory cytokines and histamine, thus aggravating the symptoms and lesions of AD [134]. In addition, it was discovered that children with AD exhibited higher levels of urine OS markers than those without AD, including 8-hydroxy-2 deoxyguanosine, nitrite, etc. [135]. Moreover, deficiency of detoxification systems and accumulation of free-radical oxidation products emerged from AD skin lesions, promoting the progress of local OS that in turn exacerbated AD attack [136].

Recent studies both *in vitro* and *in vivo* have demonstrated that flavonoids may be potentially useful in treatment of AD due to their potent antioxidant activity. *Quercetin*, as the typical representative of flavonoids, could effectively alleviate AD symptoms and AD-like lesions by combating OS and inflammation through stimulating the Nrf2/HO-1 signal and ARE depended-gene expression in AD, as well as inactivating ERK1/2 and NF-κB pathways [42, 137]; at the same time, *quercetin* had excellent abilities to AD improvement through suppressing the release of high mobility group box 1, receptor for advanced glycation end products, NF-κB and inflammatory cytokines (like IL-4, IL-1 and TNF-α) [137]. On the AD models induced by dinitrochlorobenzene (DNCB) or lipopolysaccharide-stimulated RAW264.7 macrophages, likewise, *quercetin* and *galangin* were quite a useful to ameliorate the symptoms of AD in mice via curbing ERK1/2 and JNK pathways, downregulating iNOS activity and lessening NO production [138]. Via activating Nrf2 signal, *quercetin* also availably resisted OS aggression and reduced inflammatory response induced by house dust mites in AD mice [139]. A new flavonoid, genkwanin 5-O-xylosyl (1→2) glucoside namely *stechamone* at 0.5% concentration was externally applied on the dorsal skin of AD-like murine models for 14 days, which showed that *stechamone* dramatically relieved AD-like skin manifestations, including pruritus, erythema, dryness, and lichenification, and mitigated the histopathological alterations like stratum corneum thickening, lymphocyte infiltration and mast cell degranulation, probably through fighting OS, diminishing inflammatory responses and repairing skin barrier [140]. Studies *in vitro* and *in vivo* revealed that topical application of *ISO-1*, an *isoflavone* extract from soybean, obviously alleviated DNCB-induced skin changes (e.g. erythema, trans-epidermal water loss, skin thickening and leukocyte infiltration) in DNCB-induced AD mice models, through modulation of the p38, JNK or NF-κB pathways and resistance to OS [141]. Moreover, increasing reports have demonstrated that *silymarin pluronic-lecithin organogel* could excellently lighten the inflammation, swelling and redness of AD-like lesions in AD mice, possibly being related to its inhibition of mast cell infiltration, control of OS aggression [142]. Additionally, *puerarin*, one of main isoflavone compounds, efficiently lowered the levels of cytokines and chemokines in TNF-α/IFN-stimulated HaCaT cells, and inhibited the activation of MAPKs (p38, ERK and JNK) and NF-κB signal pathways as well as STAT-1 in AD models, furthther indicating the protection of *puerarin* on AD-like skin from inflammation and OS via mediating the phosphorylation of MAPKs, NF-κB and STAT-1 [143].

Above findings suggest that flavonoids could control OS and inhibit inflammation by reducing the damage to cell macromolecules and regulating redox-related signaling pathways, providing a theoretical basis for flavonoids in the treatment of AD.

#### 3.2.8. Flavonoids in pemphigus

Pemphigus, belonging to autoimmune bullous skin disease, is caused by autoantibodies and characterized by bulla on skin and mucosa. Currently, it has been revealed that OS is a crucial player in the pathogenesis of pemphigus, regardless of MDA or LPO significantly ascending in PV patient serum and skin lesions [23, 144]. Studies have shown that the inflammatory signals in pemphigus stimulate the activation of neutrophils and the release of ROS, thereby decreasing the activity of antioxidant enzymes in plasma and erythrocyte; in turn, the reduction of antioxidants aggravates OS [145, 146]. OS further facilitates LPO and an increase in inflammatory cytokines, eventually disrupting the dermal-epidermal connection and accelerating bullous formation [144].

In recent years, several studies have demonstrated that flavonoids serve their functions thoroughly in the treatment of pemphigus. An experiment *in vitro* showed that *naringenin* could significantly reduce the production of ROS in keratinocytes of PV and relieve the decline of mitochondrial membrane potential; moreover, *naringenin* could not only enhance the levels of total antioxidant capacity, SOD and GSH-Px, but also inhibit nucleotide-binding oligomerization domain 2-mediated NF-κB pathway to protect HaCaT cell from OS injury or apoptosis induced by PV serum [147]. In another experiment basing on the Hailey-Hailey disease (HHD, a familial benign pemphigus) model, it was found that *kaempferol* effectively worked on HHD model via directly reducing OS of *ATP2C1* gene defective keratinocytes and restraining the sensitivity to ROS, further to restore mitochondrial function; at the same time, *kaempferol* could activate the Nrf2 pathway and induce the downstream target NAD(P)H: quinone oxidoreductase 1 to mitigate OS damage and protect the skin cells [148]. Recently, *Cassia fistula*, a herbal drug containing flavonoids, has been discovered to be an excellent remedy of wound healing in PV due to its oxidation resistance, the underlying mechanism being associated with DPPH and hydroxyl radical reduction [149]. However, further studies are needed to confirm the exact effect of flavonoids on pemphigus.

## 3. Conclusion

**Figure 2.**
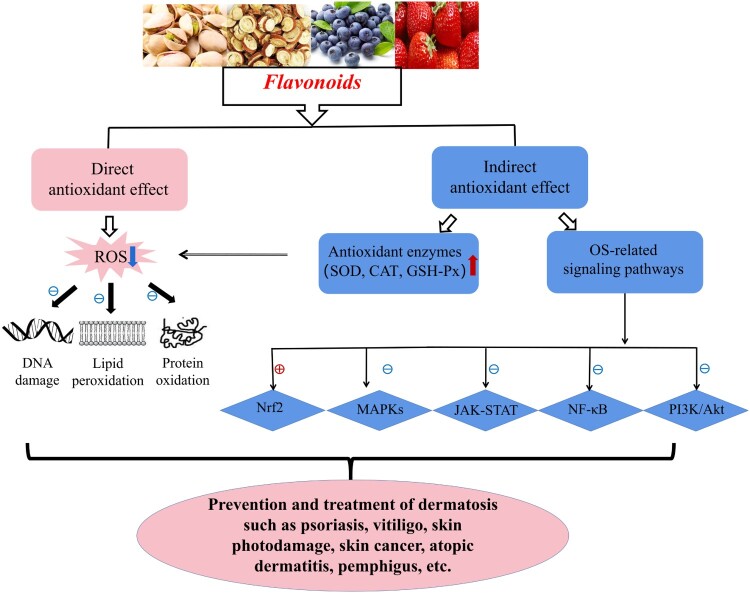
Flavonoids in control of OS-related skin diseases via multiple molecular mechanisms. Flavonoids effectively suppress OS via their direct antioxidant effects (scavenging ROS to repair DNA damage and prevent LPO and protein oxidation) and indirect antioxidant effects (reducing ROS though upregulating SOD, CAT and GSH-Px, and regulating Nrf2, MAPKs, NF-κB, JAK-STAT, PI3 K/Akt pathways), further to prevent and treat OS-related skin diseases like psoriasis, vitiligo, skin photodamage, skin cancer, SSc, chloasma, AD, pemphigus, etc. Notes: ⊕ indicates ‘ activation’; ⊖ indicates ‘inhibition or suppression’; red arrow ↑ indicates ‘up-regulation’; blue arrow ↓ indicates ‘down-regulation’.

Taken together, OS originated from a redox imbalance may cause a great damage to diversified cells/tissues, skin in particular, via several molecular mechanisms. It is closely involved in the occurrence and development of numerous skin diseases. Flavonoids, as natural plant extracts from a wide range of sources, offer a great development prospect and attract an extensive attention owing to their powerful bioactivities and few toxic side effects. Based on the strong antioxidant capacity, flavonoids may be quite effective in the prevention and treatment of various OS-related dermatoses through repairing damaged macromolecules and regulating multiple OS-associated signaling pathways (shown in Figure 2). Therefore, further studies are needed to clarify the specific mechanism as well as the dosage of flavonoids in treating different OS-related skin disorders, which would facilitate them application in clinical practice for more dermatoses recovery.
